# Core open science practices in major medical journals: development of an automatized tool based on a Large Language Model

**DOI:** 10.12688/openreseurope.24615.1

**Published:** 2026-09-07

**Authors:** Constant Vinatier, Guillaume Freyermuth, Margaux Millour, Gauthieu Le Bartz Lyan, Sarah Buet, Perrine Lunel, Nicolas Robillard, Florian Naudet, Mathieu Acher

**Affiliations:** 1 Univ Rennes, CHU Rennes, Inserm, EHESP, Irset (Institut de recherche en santé, environnement et travail) – UMR_S 1085, Rennes, France; 2 Univ Rennes, IRISA, Inria, CNRS, F-35000 Rennes, France, Rennes, France; 3 University of Rennes Faculty of Medicine, Rennes, Brittany, France; 4 Institut Universitaire de France, Paris, France

**Keywords:** open science, reproducibility, large language models, automated monitoring, transparency, medical journals, meta-research, text mining.

## Abstract

**Background:**

Open science practices are increasingly promoted to improve the transparency and reproducibility of biomedical research, and their large-scale monitoring has been identified as a priority by initiatives such as the UNESCO Recommendation on Open Science. Existing automated tools cover only narrow subsets of practices. We developed and validated an automated tool, based on locally run open-weights large language models (LLMs), to extract a core set of 13 open science practices from published medical research articles.

**Methods:**

We built a validation database of 303 research articles published in 2020–2021 in 10 major general medical journals, stratified across randomized controlled trials (RCTs), meta-analyses, and other designs. Duplicate manual extraction with expert adjudication served as the reference standard. The pipeline combined document conversion (GROBID), BM25-based chunk retrieval, and classification by three Llama models (3-8B, 3-70B, 3.3-70B) run entirely locally, with and without supplementary materials. Diagnostic performance (sensitivity, specificity, predictive values, F1) was computed against the reference standard.

**Results:**

Usable outputs were obtained for 301/303 articles. Performance was heterogeneous across criteria rather than across models: practices reported in standardized statements (conflicts of interest, funding, author contributions, data sharing, pre-registration, reporting guidelines) were extracted reliably (F1 ≥ 0.75 for at least one model), whereas rare or inconsistently reported practices (code sharing, statistical analysis plan, protocol sharing, ORCID, open access status, preprints) remained difficult regardless of model size. Study-type classification exceeded 0.90 on all metrics. Larger models improved several criteria at a 6.6–7.5× computational cost, and adding supplementary materials did not improve performance, mainly due to conversion failures.

**Conclusions:**

A fully local, open-weights LLM pipeline can reliably monitor a substantial subset of open science practices. Document conversion and layout heterogeneity, rather than language understanding, constitute the main barrier to scaling automated open science monitoring.

## Introduction

The reproducibility of scientific findings has emerged as one of the most pressing concerns in contemporary research. ^1^ A landmark analysis by Ioannidis (2005) demonstrated that the majority of published research findings might be false, attributing this to small sample sizes, high rates of false-positive results, widespread flexibility in statistical analyses and the lack of transparency. ^2^ This diagnosis was corroborated by large-scale replication initiatives, including the Reproducibility Project in Psychology, the Many Labs studies or the institute for Replication which revealed that a substantial proportion of published results could not be reproduced under comparable conditions. ^3– 5^ Open science has been proposed as a systemic response to these shortcomings. By making the products of research (data, code, protocols, preprints, …) openly accessible and verifiable, open science practices are designed to increase the transparency and reproducibility of the scientific process. ^6^

The importance of actively monitoring these practices has been recognized at the highest levels of scientific governance. The Hong Kong Principles advocate for researchers to be evaluated based on the transparency and openness of their work. ^7^ UNESCO’s 2021 Recommendation on Open Science and the G7 Science, echoed by initiatives like the Open Science Monitoring Initiative (OSMI), have similarly underscored monitoring as a priority. ^8, 9^ A consensus is also emerging on which practices should be tracked: a core set of 19 open science practices has been proposed as reference indicators for systematic monitoring in biomedicine, based on a Delphi study. ^10^

Recent advances in large language models (LLMs) offer new opportunities to automate this monitoring at scale. Early work has shown that LLMs can perform tasks such as CONSORT checklist verification with performance approaching that of human reviewers, ^11^ suggesting considerable promise for extraction of transparency indicators from the published literature.

Several tools already address specific aspects of open science monitoring. Rtransparent uses rule-based text mining to detect data sharing statements and conflict of interest disclosures, ^12^ while ODDPub targets data and code sharing using keyword-based classifiers. ^13^ However, these tools each cover a narrow subset of practices and lack the flexibility to be extended to a comprehensive monitoring framework. No existing solution provides a unified, validated pipeline capable of automatically extracting a broad set of open science indicators across heterogeneous journal formats.

The present study aims to address this gap. Building on the core set of 13 open science practices and recent advances in LLM-based information extraction, we developed and validated an automated tool for monitoring open science practices in major medical journals. Beyond, this development study, an independent validation study was conducted using a different dataset to compare the assessments generated by the final product with those obtained using existing tools designed to evaluate each practice. ^14^

## Methods

### Open science practices

The protocol was registered before the start of any analysis on the Open Science Framework (OSF) ^15^ on 24 May 2024. All data and codes generated to realize the analysis are openly available on OSF. ^15^ We used the TRIPOD LLM reporting guideline. ^16^ LLM inference was run on 2 GPUs, 96 CPU cores, and 256 GB RAM. All the needed code to reproduce the extraction are available. ^17^

***Creation of the validation database***

We included research articles published between January 1, 2020 and December 31, 2021 in 10 major general medical journals: Annals of Internal Medicine, BMC Medicine, The BMJ, The Canadian Medical Association Journal (CMAJ), JAMA, JAMA Network Open, The Lancet, Nature Medicine, The New England Journal of Medicine (NEJM), and PLoS Medicine.

All types of research articles were considered for inclusion and stratified into three categories: randomized controlled trials (RCTs), meta-analyses, and other research articles. We aimed to build a validation database equally distributed across journals and study types, targeting 10 articles per study type and per journal, for a total of 300 articles. To achieve this, a dedicated PubMed search algorithm was developed for each study type and validated according to the PRESS guidelines ^18^ by an information specialist (X.C-H.), all details are present in web appendix 1. Within each journal–category stratum, successive random draws were performed until the target of 10 articles was reached. If a selected article was found to be misclassified upon review, a new random draw was performed to replace it. In the event that a journal did not publish articles of a given study type, the remaining slots were filled by random draw from all other eligible journals, to maintain an equitable distribution across study types.

***Extracted practices***

For each article, a predefined subset of 13 open science practices was extracted, selected from the core set of 19 indicators established by community consensus using a Delphi approach. ^10^ The description and rationale for excluding/merging the remaining six indicators is detailed in Table 1. These included: preregistration (yes/no); data sharing (fully open access, available on request, or not available); open access publishing (yes/no); code sharing (yes/no); materials sharing, including study protocol and statistical analysis plan (yes/no); use of a reporting guideline (yes/no, and if yes, which one); publication of results within one year of study completion, applicable to RCTs only (yes/no); preprint availability (yes/no); author contribution statement (yes/no); conflict of interest statement (yes/no); ORCID identifier reporting (yes/no); and funding statement (yes/no). In addition to these items, study type classification (RCT, meta-analysis, network meta-analysis, or observational study) was extracted as a supplementary variable to evaluate the ability of the LLM to detect it.

**Table 1.** Description of the 12 Traditional Open Science practices and the 7 Broader transparency practices obtained from a Delphi study ^10^ and decision to include it or not in the observatory with the appropriate prompt used for each criteria.

Open Science Practices	Inclusion in the study (Label)	Explanation & Elaboration	Prompt	Scoring
**Items included**				
Reporting whether clinical trials were registered before they started recruitment	Yes (Registration RCT)	As automated tools cannot determine from the paper alone whether registration was prospective, we only verified whether a registration record existed.	Were the study clinical trials registered in a registry such as NCT, EU-CTR, ChiCTR or PROSPERO for example? Answer with YES or NO. Answer only with a single word.	YES = 1, NO = 0
Reporting whether author contributions were described	Included (Contribution statement)	We assessed whether the article included a contribution statement.	Are the roles of the authors involved in this study mentioned? Answer with YES or NO. Answer only with a single word.	YES = 1, NO = 0
Reporting whether research articles include funding statements	Included (Funding statement)	We monitored whether the article included a funding statement.	Is there a statement that specifies fundings and grants received by the authors or for the study? Answer with YES or NO. Answer only with a single word.	YES = 1, NO = 0
Reporting whether author conflicts of interest were described	Yes (COI)	We assessed whether the article included a conflict-of-interest disclosure.	Is there a declaration of interest or conflicts of interest, like the role of the funders or the affiliations of the authors? Answer YES if a statement mentions no competing interest. Answer YES if the study mentions there is no conflict declared. Answer NO if the COI statement says it is not applicable or not filled. Answer with YES or NO only.	YES = 1, NO = 0
Reporting whether study data were shared openly at the time of publication (with limited exceptions)	Yes (intention to share data)	Given the sensitivity of biomedical research data, we defined intention to share data broadly as any declared intention to share data, whether openly or upon request. These estimates may therefore overstate actual data sharing, as authors may refuse requests.	Is there a data sharing statement specifying how the anonymised patient data are shared? Answer 1 if the data are accessible without a request, 2 if accessible on request, 0 otherwise. Do not justify your answer.	1 = open, 2 = on request, 0 = none
Reporting what proportion of articles are published open access with a breakdown of time delay	Yes (Open access publishing)	For this item, automated extraction tools are unsuitable because the relevant information is usually not contained within the article text. We therefore relied on available metadata.	Is it clearly mentioned if this study is Open Access (OA)? Answer with YES or NO. Answer only with a single word.	YES = 1, NO = 0
Reporting whether ORCID identifiers were used	Yes (ORCID)	We assessed whether the article included at least one ORCID identifier.1	Does this study contain ORCID identifiers? Answer with YES or NO. Answer only with a single word.	YES = 1, NO = 0
Reporting whether systematic reviews have been registered before data collection began	Yes (Registration MA)	As automated tools cannot determine from the paper alone whether registration was prospective, we only verified whether a registration record existed.	Were the study clinical trials registered in a registry such as NCT, EU-CTR, ChiCTR or PROSPERO for example? Answer with YES or NO. Answer only with a single word.	YES = 1, NO = 0
Reporting whether a reporting guideline checklist was used	Yes (Reporting guideline)	We assessed whether reporting guidelines were mentioned, but we did not evaluate reporting quality. This may underestimate actual use, as we only detected instances where reporting guidelines were explicitly cited or referenced.	Does this study mention a specific reporting guideline, such as PRISMA, GATHER, CONSORT, STROBE, TORIPOD, CHEERS, MOOSE, ENTREQ, CHARMS, RECORD, AAPOR, COREQ or TIDieR? Answer with YES or NO. Answer only with a single word.	YES = 1, NO = 0
Reporting whether study code was shared openly at the time of publication (with limited exceptions)	Yes (Code sharing)	As with intention to share data, we used a broad definition that included both open access and on-request access. These estimates may overestimate code sharing, as authors may deny requests.	Is there a data sharing statement specifying that this study shares code snippets to reproduce the study (R, Python, etc.)? Answer 1 if code is accessible without a request, 2 if accessible on request, 0 otherwise. Do not justify your answer.	1 = open, 2 = on request, 0 = none
Reporting the number of preprints	Yes (Pre-Print)	We tracked references to preprints within the article.	Does the article mention the existence of a preprint version? Answer with YES or NO. Answer only with a single word.	YES = 1, NO = 0
Reporting whether clinical trials results appeared in the registry from 1 year after study completion	Yes (publication 1 year after the end of the study (for RCT))	To establish this, we employed either the date of the final visit for the last patient or the inclusion end date in combination with the specified follow-up duration.	Was this study published within 1 year of the last visit of the last patient? Answer with YES or NO. Answer only with a single word.	YES = 1, NO = 0
**Items not included**				
Reporting whether there was a statement about study materials sharing with publications	No	Due to substantial heterogeneity in what constitutes material sharing across biomedical research, it was not feasible to monitor this item consistently across all articles.		
Reporting citations to data	No	This item was considered too similar to “Intention to share data” and too difficult to apply consistently to current publications.		
Reporting trial results in a manuscript-style publication (peer reviewed or preprint)	No	All published journal articles inherently meet this criterion; therefore, monitoring it within journal publications was deemed unnecessary.		
Reporting systematic review results in a manuscript-style publication (peer reviewed or preprint)	No	All published journal articles inherently meet this criterion; therefore, monitoring it within journal publications was deemed unnecessary.		
Reporting whether data/code/materials are shared with a clear license	No	This item was excluded because it is challenging to evaluate it using only the content of current research articles.		
Reporting the use of persistent identifiers when sharing data/code/materials	No	This item was excluded because it is challenging to evaluate it using only the content of current research articles.		
Reporting whether the data/code/materials license is open or not	No	This item was excluded because it is challenging to evaluate it using only the content of current research articles.		

***Manual data extraction***

Data collection was carried out by three first-year Master’s students (SB, PL, NR), who were first trained on a calibration sample of 10 articles. In case of doubt during extraction, they could consult C.V. or F.N. (domain experts) to ensure consistency. Owing to calendar constraints, all manual extraction was completed before registration of the study. However, no automated analysis was performed before registration. All articles and supplementary materials were retrieved from PubMed and from the journals’ websites; for articles not available in open access, institutional academic access was used. All data were extracted independently and in duplicate. In case of disagreement between evaluators, a final adjudication was performed by C.V. Manual extraction served as the reference standard to evaluate the performance of the developed tool.

### Ethics statement

This study did not involve human participants or personal data: it consisted exclusively of the analysis of previously published research articles, and all information was extracted from the published literature by members of the research team (SB, PL, NR), who are co-authors of this article. In accordance with national and institutional guidelines, ethical approval and informed consent were therefore not required for this study.

### Automatic extraction

We developed an automated classification pipeline comprising three sequential steps: document conversion, chunk retrieval, and LLM-based classification. Document conversion: Research articles in PDF format were first converted into a machine-readable format using GROBID v0.8.2, ^19^ run via its official Docker image (lfoppiano/grobid:0.8.2) with Apptainer. GROBID extracts text while preserving the natural reading order of the document and associated metadata, producing a TEI XML output. The extracted text was then segmented into overlapping chunks, each providing sufficient context for the model to answer a given question without exceeding its optimal input length.

The supplementary files were processed using a separate extraction workflow adapted to each file format: standard Python I/O functions for *.txt files, the python-docx library for *.docx files, and PyMuPDF (fitz) for *.pdf files. Given the large size and heterogeneous content of supplementary PDFs, a filtering step was applied to retain only pages where text accounted for more than 50% of the content relative to other elements such as tables and images. This step was necessary due to computational constraints, although we cannot exclude that it may have resulted in some data loss for highly visual supplementary materials.

Chunk retrieval. The converted text was segmented into overlapping chunks of 256 tokens with a 32-token overlap. For each classification question, the 8 most relevant chunks were retrieved using BM25, ^20^ a lexical ranking function based on term frequency, and provided to the LLM as context. In pilot comparisons conducted during development, a dense-retrieval alternative (chunk embeddings generated with the Microsoft BiomedNLP-BiomedBERT model and stored in a ChromaDB vector database) showed no overall benefit: BM25 outperformed it on two of the three criteria tested (data sharing and conflicts of interest), with study type as the only exception. BM25 was therefore adopted for all criteria, and all results reported here were obtained with BM25 retrieval.

LLM-based classification. The model was prompted with the retrieved context and a classification question for each outcome. To ensure structured outputs, the model was instructed to respond with a single word (YES/NO) or a single digit (0, 1, 2, or 3), and responses were parsed accordingly. If no valid answer was detected, a predefined default value was applied, specific to each criterion. The prompts used for each outcome are provided in Table 1.

**Figure 1.**
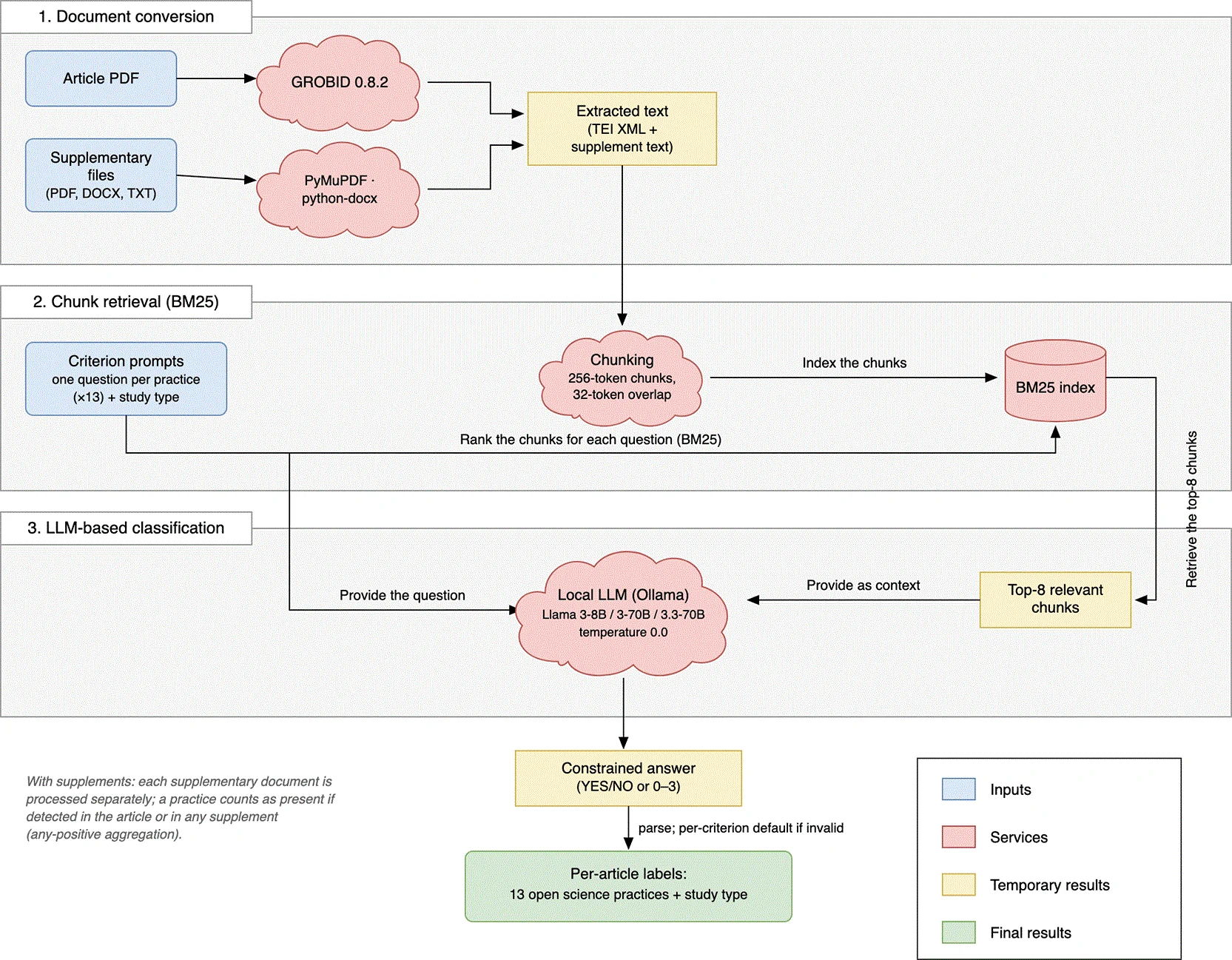
Schematic representation of the three-step LLM-based pipeline for automated extraction.

The pipeline was built around open-source LLaMA models with publicly available weights running locally, ensuring full reproducibility without reliance on external APIs, and guaranteeing that documents were not shared externally due to copyright obligations. Three models were used to perform the automated extraction, Llama 3 8B, Llama 3 70B and Llama 3.3 70B, allowing comparison of performance across model sizes. To maximize reproducibility across runs, all inferences were performed with a temperature of 0.0. For each model, the extraction pipeline was run separately on the main article text and on the supplementary materials, in order to assess whether supplements provided additional information not captured in the main text; an open science practice was considered present if it was identified in either source. None of the three models was fine-tuned, as our dataset served exclusively as a validation set; fine-tuning on it would risk overfitting without guaranteeing generalizability. Figure 1 details the overall pipeline.

### Prompt and pipeline development

The pipeline and prompts were developed iteratively from June to August 2024, jointly by a clinician meta-researcher (CV) and the engineering team, before the final evaluation runs. Alternative designs were explored and discarded during this phase: rule-based (regular-expression) classification, passing the full article text as context, paragraph-level chunking with general-purpose embeddings, LLM-based query expansion, and neural reranking (each abandoned for insufficient recall or prohibitive computational cost). Prompts were refined over several iterations informed by qualitative error analysis against the manual annotations: answer formats were constrained to a single word or digit to make parsing deterministic; edge cases were made explicit (e.g., counting “no competing interests” statements as a positive conflict-of-interest disclosure). Moreover, the reporting-guideline prompt enumerates common guideline names. Variants asking the model to justify its answers were tried and abandoned, as requesting a justification altered the answers and increased processing time; automatic keyword mining (decision trees over word-count representations) to enrich queries yielded useful terms only for study type. The complete development log is available in the code repository, and all prompts (including abandoned variants) are provided.

### Statistical analysis

A descriptive analysis was conducted on articles that were manually extracted from the validation database, described by the percentage of presence for each practice.

The tool’s performance was determined by computing sensitivity, specificity, positive predictive value (PPV), negative predictive value (NPV) and the F1 score against the manual reference standard, bearing in mind that disagreements may also stem from errors in the human extraction. ^21^ For each disagreement, a manual verification of the result by an expert (CV) was done to determine if the LLM is truly false or not, for data sharing, reporting guidelines, and registration.

All statistical analysis and data visualisation has been realised using R Project for Statistical Computing (RRID: SCR_001905) version 4.5.2, with the libraries tidyverse (RRID: SCR_019186). All analyses were performed on a MacBook Pro with an M5 chip.

### Modifications to the protocol

Protocol was mentioning the plotting of the Receiver Operating Curve; this was not done, because the method of classification used (boolean or categorial output, not associated with a probability) was not suitable for this type of plot.

We additionally tested whether including supplementary materials in the extraction pipeline improved or impaired performance, compared to using the main article text alone. Supplementary files were processed as independent documents alongside the main article text; when a given open-science practice was detected as positive in either the main text or in any supplementary document, the article was labelled positive (conservative disjunctive aggregation).

Initially, all negative LLM outputs were planned to be verified by a human evaluator; however, this was ultimately limited to a subset of criteria considered easily detectable yet showing poor model performance, corresponding to 66 articles for data sharing, 60 for reporting guidelines, and 55 for pre-registration.

## Results

### Research articles selection

**Figure 2.**
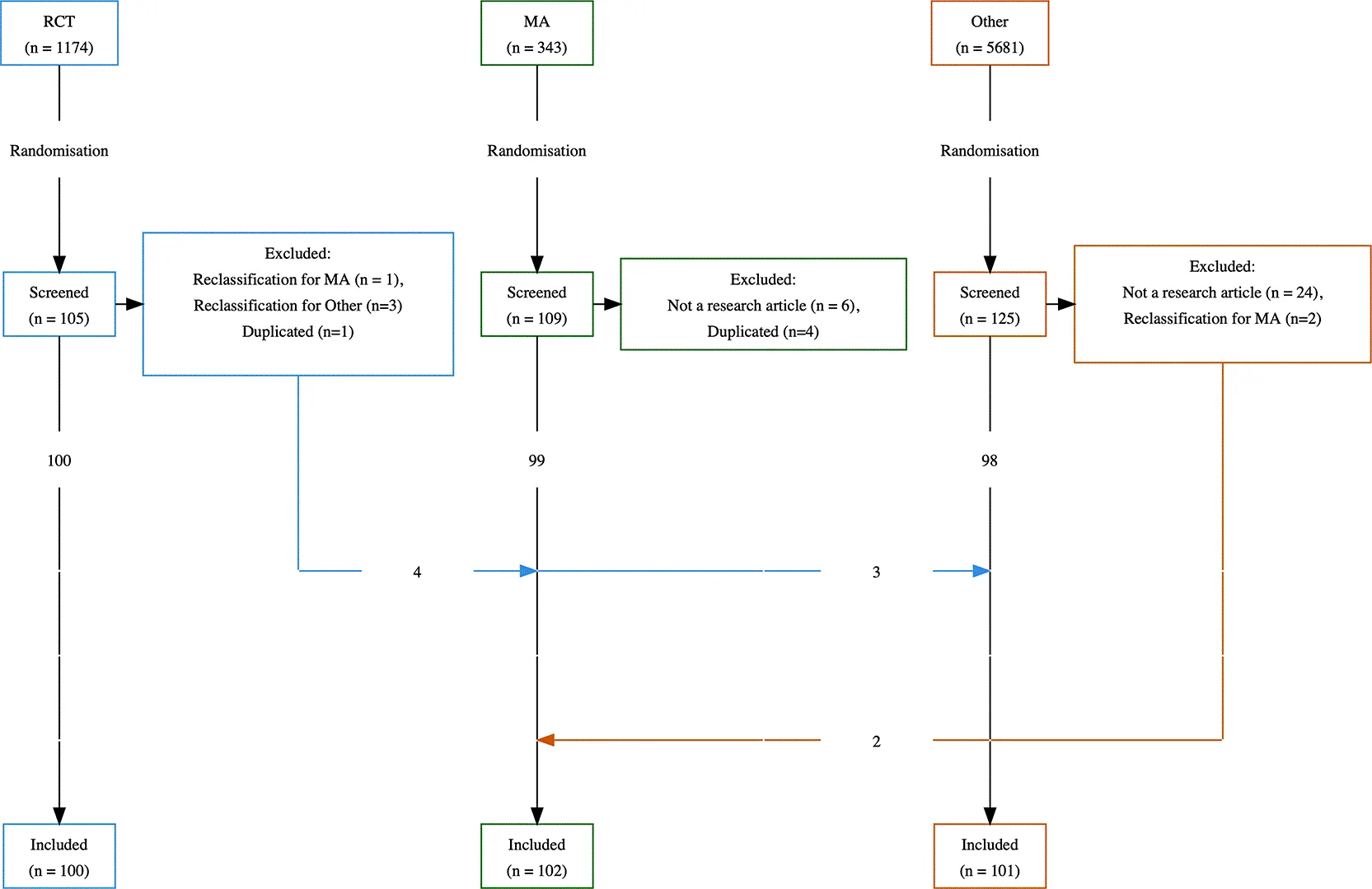
Flowchart of the database creation process. * For randomized controlled trials and meta-analyses, three separate rounds of selection were required due to reclassification and duplication issues, caused by incorrect categorization within PubMed. ** For other types of research studies, five rounds of selection were necessary due to reclassification, exclusion of one retracted article, and incorrect categorization as research articles.

PubMed searches were conducted on February 25th, 2024, with data extraction taking place between February 25th and March 28th, 2024. Figure 2 presents the study flow diagram, illustrating the selection process for the validation database, including both the articles retained and those misclassified. Due to misclassifications related to study type, several articles had to be excluded or reclassified, which necessitated multiple rounds of randomization. In total, 303 research articles were included in the validation database (100 RCTs, 102 meta-analyses, and 101 articles of other study types).

### Validation database

Not all journals published every type of research article. For instance, we found only one meta-analysis published in NEJM and only one RCT published in CMAJ. Table 2 presents the descriptive analysis of the validation database. Regarding transparency in reporting, conflicts of interest were disclosed in almost all research articles (100%), and a funding statement was reported in 95% of articles. Mention of preprints was rare, with only four identified in the validation database (1.3%). Registration (99% vs 56% vs 12%), protocol sharing (74% vs 59% vs 8.8%), and statistical analysis plan sharing (41% vs 4.0% vs 3.9%) were consistently more common in RCTs than in meta-analyses or other types of research articles. Conversely, adherence to a reporting guideline was more frequent in meta-analyses (76%) than in RCTs (28%) or other articles (28%). Code sharing remained limited across all study types, with no sharing reported in 95% of RCTs, 82% of meta-analyses, and 77% of other articles. Authors most often indicated that data were available “upon request” rather than openly, particularly for RCTs (72% vs 9%).

**Table 2.** Open science practices in the validation database obtained by manual extraction and after checks (considered as the gold standard).

Characteristic	Overall N = 303 ^1^	RCT N = 100 ^1^	MA N = 101 ^1^	RA N = 102 ^1^
Journal				
Ann Intern Med	32 (11%)	10 (10%)	11 (11%)	11 (11%)
BMC Med	33 (11%)	10 (10%)	13 (13%)	10 (9.8%)
BMJ	32 (11%)	10 (10%)	12 (12%)	10 (9.8%)
CMAJ	17 (5.6%)	1 (1.0%)	6 (5.9%)	10 (9.8%)
JAMA	33 (11%)	12 (12%)	11 (9.9%)	10 (9.8%)
JAMA Netw Open	37 (12%)	10 (10%)	17 (17%)	10 (9.8%)
Lancet	33 (11%)	13 (13%)	11 (11%)	9 (8.8%)
N Engl J Med	25 (8.3%)	14 (14%)	1 (1.0%)	10 (9.8%)
Nat Med	24 (7.9%)	10 (10%)	3 (3.0%)	11 (11%)
PLoS Med	37 (12%)	10 (10%)	16 (16%)	11 (11%)
Registration	168 (55%)	99 (99%)	57 (56%)	12 (12%)
Data Sharing				
no sharing	106 (35%)	19 (19%)	43 (43%)	44 (43%)
open access	52 (17%)	9 (9.0%)	26 (26%)	17 (17%)
under request	145 (48%)	72 (72%)	32 (32%)	41 (40%)
Open access Sharing	259 (85%)	75 (75%)	91 (90%)	93 (91%)
Code sharing				
no sharing	257 (85%)	95 (95%)	83 (82%)	79 (77%)
open access	26 (8.6%)	1 (1.0%)	11 (11%)	14 (14%)
under request	20 (6.6%)	4 (4.0%)	7 (6.9%)	9 (8.8%)
Publication 1 year RCT	21 (19%)	14 (14%)	0 (0%)	7 (78%)
Protocol				
no sharing	151 (50%)	24 (24%)	41 (41%)	86 (84%)
open access	143 (47%)	74 (74%)	60 (59%)	9 (8.8%)
under request	9 (3.0%)	2 (2.0%)	0 (0%)	7 (6.9%)
Statistical Analysis Plan				
no sharing	249 (82%)	55 (55%)	97 (96%)	97 (95%)
open access	49 (16%)	41 (41%)	4 (4.0%)	4 (3.9%)
under request	5 (1.7%)	4 (4.0%)	0 (0%)	1 (1.0%)
Reporting guideline	134 (44%)	28 (28%)	77 (76%)	29 (28%)
Preprint	4 (1.3%)	0 (0%)	3 (3.0%)	1 (1.0%)
Author contribution	281 (93%)	87 (87%)	101 (100%)	93 (91%)
COI	302 (100%)	100 (100%)	100 (99%)	102 (100%)
ORCID	120 (40%)	42 (42%)	42 (42%)	36 (35%)
Funding_statement	288 (95%)	98 (98%)	93 (92%)	97 (95%)

^1^ n (%)

The correction performed by CV after the automatic extraction identified 21 errors out of 66 articles checked for data sharing, 10 out of 60 for reporting guidelines, and 10 out of 55 for pre-registration.

### Tool performance

***Comparison across models***

**Figure 3.**
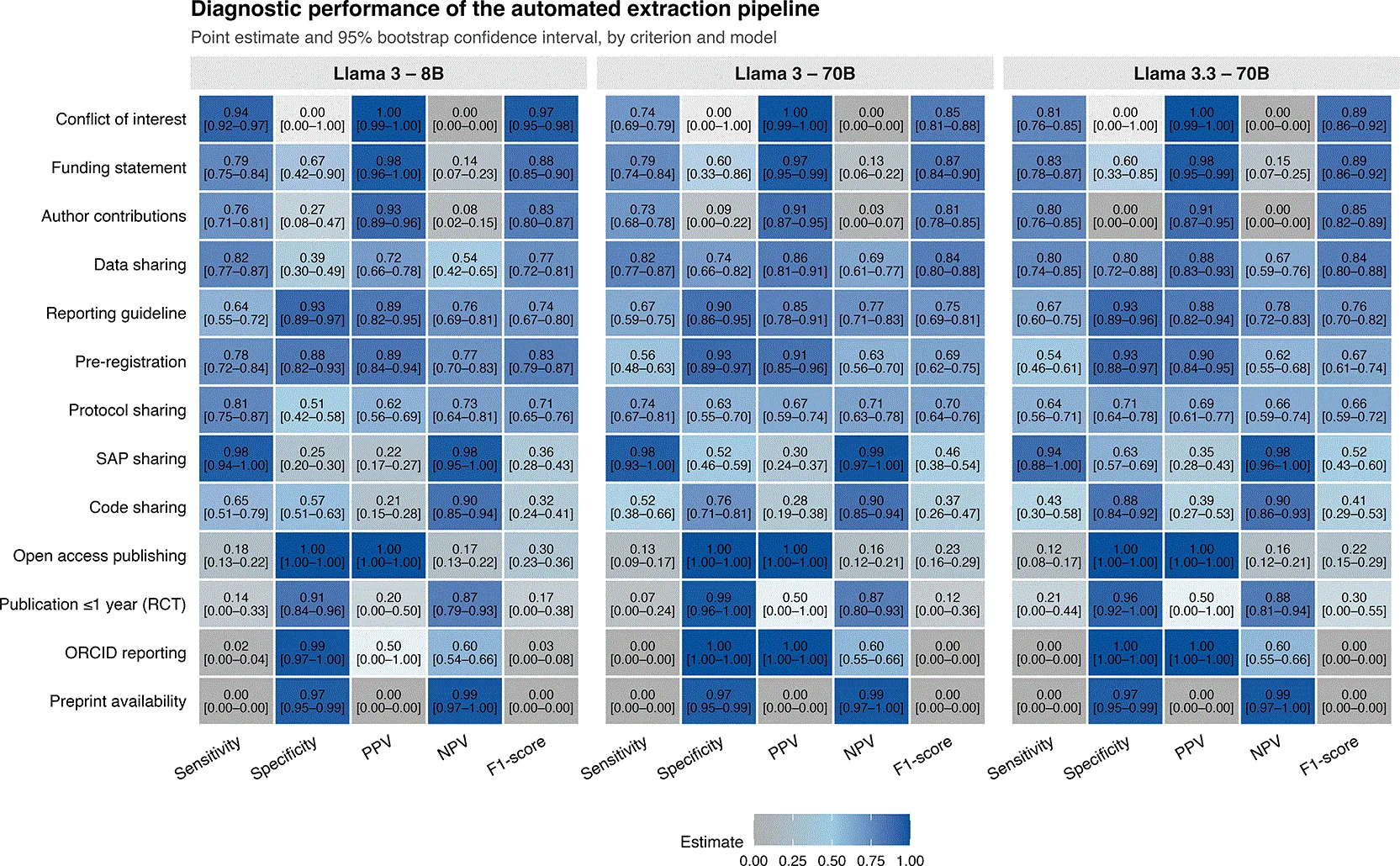
Diagnostic performance of the automated extraction pipeline. Point estimate and 95% bootstrap confidence interval, evaluated for each model and outcomes.

Of the 303 articles in the validation database, 301 (99%) yielded a usable LLM output; two articles from JAMA were excluded because document conversion by GROBID failed. Figure 3 present the performance of the automated extraction for each of the 13 open-science practices and the detection of the study type, comparing the three different models (Llama 3-8B, Llama 3-70B, and Llama 3.3-70B), using the main article text alone (without supplements). Performance varied substantially across criteria: detection of COI statements was the most reliably extracted (Llama 3-8B: sensitivity = 0.94 [95% CI 0.92–0.97], specificity = 0.00 [95% CI 0.00–1.00], PPV = 1.00 [95% CI 0.99–1.00], NPV = 0.00 [95% CI 0.00–0.00], F1 = 0.98 [95% CI 0.95–0.98]), the wide confidence interval around specificity and the null NPV reflect the near-total absence of true negatives for this criterion (COI disclosure was positive in ~100% of articles) rather than a genuinely unstable estimate. Conversely, open-access publishing status was the least reliable practice to extract across models (sensitivity = 0.12–0.18 [95% CI 0.08–0.22], specificity = 1.00 [95% CI 1.00–1.00], PPV = 1.00 [95% CI 1.00–1.00], NPV = 0.16–0.17 [95% CI 0.12–0.22], F1 = 0.24–0.33 [95% CI 0.15–0.36] across models), indicating that the tool systematically under-detected true positives while never producing a false positive.

For criteria with extreme class imbalance: COI (positive in ~100% of articles), preprint availability (positive in ~1%), and ORCID reporting, single summary metrics could be misleading taken in isolation. For ORCID, specificity remained high across all three models and PPV near-perfect, while sensitivity was close to zero, indicating the tool essentially never identified a true ORCID mention. This near-total failure on the positive class, masked by an apparently high specificity, is likely explained by the fact that ORCID identifiers are typically not expressed in the body text of the manuscript but are instead embedded as a tooltip linked to the ORCID icon displayed next to the author’s name. As such, this information was never part of the text extracted by GROBID and passed to the LLM for extraction. Similarly, for preprint availability, the diagnostics performances reflect the near-absence of true positives in the dataset rather than genuine discriminative ability.

Compared to Llama 3-8B, Llama 3.3-70B performed better on code sharing (F1 from 0.32 to 0.41), statistical analysis plan sharing (F1 from 0.36 to 0.52), author-contribution statements (F1 from 0.83 to 0.85), funding statements (F1 from 0.88 to 0.89), use of a reporting guideline (F1 from 0.74 to 0.76), data sharing (F1 from 0.77 to 0.84) and publication within 1 year for RCT (F1 from 0.17 to 0.30). Conversely, Llama 3-8B remained the best-performing model for COI disclosure (F1 = 0.97 compared to 0.85 for Llama 3-70B and 0.89 for Llama 3.3-70B) and pre-registration detection (F1 = 0.83 compared to 0.69 for Llama 3-70B and 0.67 for 3.3-70B). The three models performed equivalently for study-type classification, protocol sharing, preprint detection, and ORCID reporting. Considering computational cost, the full 13-criterion pipeline required approximately 22 minutes for Llama 3-8B versus 147 minutes (6.6×) for Llama 3-70B and 168 minutes (7.5×) for Llama 3.3-70B.

The automated classification of study type yielded high performance across all three models and all three classes (RCT, meta-analysis, other), with sensitivity, specificity, PPV, NPV, and F1-scores systematically above 0.90 ( Table 3). This consistency across both models and classes indicates that the pipeline reliably distinguishes the main study designs in the validation set, with no model emerging as clearly superior for this task.

**Table 3.** Diagnostic performances of the 3 models (Llama 3-8B, 3-70B, and 3.3-70B) to detect the type of study.

Model	Class	Sensitivity	Specificity	PPV	NPV	F1
Llama 3 – 8B	RCT	1.00 [1.00–1.00]	0.96 [0.93–0.98]	0.92 [0.86–0.96]	1.00 [1.00–1.00]	0.96 [0.93–0.98]
	MA	0.96 [0.92–0.99]	0.97 [0.94–0.99]	0.93 [0.88–0.98]	0.98 [0.96–1.00]	0.95 [0.91–0.98]
	Other	0.86 [0.79–0.92]	0.99 [0.97–1.00]	0.98 [0.94–1.00]	0.93 [0.90–0.97]	0.92 [0.87–0.95]
Llama 3 – 70B	RCT	0.99 [0.97–1.00]	0.96 [0.93–0.99]	0.92 [0.87–0.97]	0.99 [0.98–1.00]	0.96 [0.93–0.98]
	MA	0.93 [0.87–0.98]	0.98 [0.95–1.00]	0.95 [0.90–0.99]	0.97 [0.94–0.99]	0.94 [0.90–0.97]
	Other	0.88 [0.82–0.94]	0.96 [0.94–0.99]	0.93 [0.87–0.97]	0.94 [0.91–0.97]	0.90 [0.86–0.94]
Llama 3.3 – 70B	RCT	0.97 [0.93–1.00]	0.99 [0.97–1.00]	0.97 [0.93–1.00]	0.99 [0.97–1.00]	0.97 [0.94–0.99]
	MA	0.89 [0.83–0.95]	0.98 [0.96–1.00]	0.96 [0.91–0.99]	0.95 [0.92–0.98]	0.92 [0.88–0.96]
	Other	0.94 [0.89–0.98]	0.93 [0.90–0.97]	0.88 [0.81–0.93]	0.97 [0.94–0.99]	0.91 [0.86–0.95]

* RCT: Randomised control trial; MA = Meta-analysis

***Impact of including supplementary materials***

Across the 301 articles for which extraction was attempted, we retrieved a total of 543 PDF, 247.docx and 72.xlsx supplementary files. However, only 56% (483/862) of these files could be converted into machine-readable text, the remainder consisting either of scanned images, image-based tables, or files whose internal structure could not be parsed by the current pipeline. After grouping usable supplementary text at the article level, 231 of the 301 articles (77%) had at least one supplementary document providing extractable text and were therefore eligible for the with-supplement extraction condition.

Comparing the with- and without-supplement conditions, the addition of supplementary text did not produce a consistent improvement in performance for either Llama 3-8B or Llama 3-70B (web appendix 2). For Llama 3-8B, F1-scores were essentially stable across the two conditions for most criteria, with small gains observed for open-access publishing (0.30 [0.23–0.37] to 0.35 [0.28–0.42]), reporting guideline (0.74 [0.67–0.80] to 0.77 [0.70–0.82]), and author-contribution statements (0.83 [0.80–0.87] to 0.86 [0.83–0.89]). These gains, however, were partly offset by systematic drops in specificity for criteria where the tool tended to over-trigger on the noisier supplementary text, notably: protocol sharing (specificity 0.51 [0.43–0.59] to 0.32 [0.24–0.39]) and SAP sharing (specificity 0.25 [0.20–0.31] to 0.14 [0.10–0.18]), indicating that the extra recall came at the cost of additional false positives. For Llama 3-70B, the pattern was even more balanced: F1-scores changed by less than 0.05 for the majority of criteria, and no direction of effect was consistently favorable.

## Discussion

### Main findings

This study assessed the feasibility of a fully local, open-weights LLM pipeline for the automated extraction of 13 open-science practices and study-type classification across 301 articles published in 10 major medical journals. Performance was highly heterogeneous across criteria rather than across models: practices reported in a standardized, stereotyped form in the main text: COI disclosure, funding statements, author contribution, data sharing, pre-registration, reporting-guideline names, and study type; were extracted reliably (F1 ≥ 0.75 for at least one model), whereas practices that are rare, inconsistently phrased, or reported in supplementary materials: code sharing, statistical analysis plan sharing, protocol sharing, ORCID identifiers, Open access publishing and preprint availability; remained difficult regardless of model size. Increasing model scale improved performance for code sharing, statistical analysis plan sharing, author-contribution statements, funding statements, use of a reporting guideline, data sharing and publication within 1 year for RCT. COI disclosure and pre-registration detection in particular remained best handled by the smallest model, at a computational cost 5.7 to 6.6 times higher; the larger models were therefore not consistently favored. In addition, the pipeline proved highly informative for classifying study types, with no model substantially outperforming the others.

Including supplementary materials in the retrieval pipeline did not meaningfully change performance for any criterion when tested with Llama 3-8B and with Llama 3.3-70B.

### Heterogeneity among the source documents

A central obstacle encountered throughout this project was the heterogeneity in how the journals format the main text and the supplementary files. Depending on the open-science practice considered, each journal has its own way of communicating it considerably complicating the generalizability of LLM-based extraction. ^13^ For instance, in our validation database, we observed that data and code-sharing statements are displayed by JAMA-family journals and NEJM in a dedicated supplementary file, by PLOS in a dedicated column parallel to the introduction, and by Annals of Internal Medicine in a “Reproducible Research Statement” placed at the end of the article. Enabling standardized monitoring would require open-science statements to be reported in a standardized location, both to facilitate information extraction and to ensure that the relevant information is reliably conveyed to the LLM for analysis.

This heterogeneity was compounded for supplementary materials, which are rarely standardized in format (PDF, Word, plain text, or scanned images) and frequently combine narrative text with tables, checklists, and checkbox grids. ^22^ Journals generally enforce a standardized presentation for the main manuscript, but supplementary files are typically not subject to the same editorial review, and the lack of a harmonized presentation across and even within journals creates additional heterogeneity. ^22^ Moreover, supplementary files often consist primarily of tables, figures, and secondary analyses that are not extractable as plain text and therefore cannot be reliably analyzed by an LLM. ^23^ This issue is particularly acute when supplementary information is provided as scanned images or screenshots rather than as machine-readable text: in such cases, GROBID, like most text-extraction tools used in this pipeline, cannot recover any content at all, since it operates on the document’s text layer and has no optical character recognition capability. ^19^ This is consistent with our finding that adding supplementary materials did not improve extraction performance: the information is often present in the source document, but is lost at the conversion phase before it ever reaches the language model. This points to document-layout and table extraction, rather than language understanding, as the primary methodological barrier to scaling this kind of monitoring across journals with heterogeneous publishing conventions.

### Relevance of humans as a ‘gold standard’

We used double independent extraction by trained evaluators with expert adjudication of disagreements as the reference standard against which the LLM pipeline was evaluated. However, framing this human-derived dataset as a true “gold standard” deserves some nuance. A methodological review of data extraction in systematic reviews found that single-reviewer extraction error rates typically range from 15% to over 40%, and that even double extraction (standard recommended by Cochrane) does not eliminate errors, only reduces their frequency relative to single extraction. ^24^ In our study, we applied this gold-standard approach using two independent reviewers, yet residual errors were still observed, consistent with the limitations described in the literature. Disagreements between reviewers most often arise not from simple transcription mistakes but from differing interpretations of what counts as a positive instance of a given criterion. Our own experience supports this: the adjudication process itself revealed that a sizable fraction of initial annotations for some criteria (notably data sharing, where roughly a third of audited “data sharing” labels were corrected) reflected disagreement about definitions rather than oversight. This situation closely parallels the broader debate in evidence synthesis about what a “gold standard” data extraction actually represents: as in systematic reviews and meta-analyses, where dual extraction is recommended precisely because no single human reading of a paper is considered infallible, our reference dataset should be understood as the best available approximation of the truth rather than an error-free ground truth. This has two consequences for interpreting our results: first, part of the apparent disagreement between the LLM and the reference standard likely reflects genuine ambiguity in how a practice is reported, not only a tool failure; second, recently developed LLM-assisted “second-reviewer” approaches to data extraction — shown in early comparative work to perform comparably to, though not yet able to fully replace, human extraction ^25^ — suggest that the most realistic short-to-medium-term role for tools like ours may be as a complement to, rather than a replacement for, human curation.

### Limitations

Several limitations should be acknowledged. First, the language models evaluated here are not the latest and the most capable LLMs currently available for text-classification tasks; substantially larger proprietary models accessed through commercial APIs would plausibly achieve higher performance on at least some criteria. Our choice of using local models was deliberate rather than incidental: articles processed in this project, and more generally in the literature, are protected by copyright and might be distributed under paywalled access agreements that explicitly prohibit redistributing full text to third parties, a category that, can reasonably be argued to include sending full-text content to an external commercial API whose data-retention and reuse policies are often not fully transparent or auditable. Moreover, as it’s unsure how a model is truly trained, this approach enables reproducible results. Running an open-weights model entirely locally avoids this legal and confidentiality exposure by design, at the cost of using models that are somewhat behind the state of the art.

Second, this choice also has an environmental dimension: inference, not training, is now understood to be the dominant contributor to the lifecycle energy footprint of large language models, and that footprint scales roughly with model size and the volume of queries processed. ^26, 27^ Running repeated full-text classification queries against very large models hosted on remote GPU infrastructure for a corpus of thousands of articles is not environmentally neutral. Moreover, the poor performance observed for some criteria reflects not a limitation of the LLM itself but the absence of the relevant information in the article text. This is most apparent for open-access publishing status or the fact to have created a preprint, which is rarely stated explicitly in the manuscript and is instead documented in publisher metadata or licensing records; for such criteria, services such as Unpaywall would achieve substantially better performance at a fraction of the computational cost. ^28^ Finally, given the rapid pace at which new LLMs are released, the relative performance observed here may shift as more capable models become available; the lasting contribution of this work therefore lies less in the specific models evaluated than in the methodological framework and the upstream bottlenecks it identifies. Relatedly, although none of the models was fine-tuned, prompts were refined during development using qualitative error analysis against the manual annotations. A further limitation is that the pipeline could only be evaluated end-to-end, which prevents us from attributing errors to a specific component: an incorrect classification may result from GROBID failing to extract the relevant section of text, from the retrieval step failing to surface the most pertinent passages to the LLM, or from the LLM itself failing to answer correctly despite being given the relevant information in context. A component-wise evaluation would be needed to disentangle these three potential failure modes and guide targeted improvements of the pipeline.

## Conclusion

In light of these results, we retained Llama 3.3-70B for use, accepting its higher computational cost in exchange for its overall best per-criterion performance; supplementary materials were also kept in the pipeline as a conservative choice, despite their negligible measured impact. Beyond this validation, the manually curated database may serve as a benchmarking resource for future automated extraction tools, complementing the independent 2022–2023 validation set used in our companion study. ^14^

## Software and code availability

Software available from: https://gitlab.inria.fr/glebartz/mr_oaso

Source code available from: https://gitlab.inria.fr/glebartz/mr_oaso

Archived source code at time of publication: The exact version underlying this article has been permanently archived on Zenodo, https://doi.org/10.5281/zenodo.21704471. ^29^

License: The source code is released under the MIT License.

The complete source code of the extraction pipeline, including all prompts and the development log, is openly available and version-controlled at the repository above.

## Ethics and consent

Ethical approval and consent were not required, as this study did not involve human participants and was based exclusively on previously published research articles.

## AI statement

The authors used Claude (Opus 4.8 or Fable5, Anthropic PBC, San Francisco, CA) to assist with language editing of the manuscript — specifically to check grammar, improve clarity, and refine phrasing of selected sections of the main text. The tool was not used to generate scientific content, analyse data, or produce results. All AI-assisted text was reviewed and verified by the authors, who take full responsibility for the integrity and accuracy of the entire content of the manuscript.

## Data Availability

Open Science Framework: Open science policies and core open science practices in major medical journals: development of an automatized tool based on a LLM. DOI: https://doi.org/10.17605/OSF.IO/T4RXC. ^15^ This project contains the following underlying data: the validation database of the 303 manually extracted research articles (duplicate manual extraction with expert adjudication, used as the reference standard), the outputs of the automated extraction for each model (Llama 3-8B, Llama 3-70B and Llama 3.3-70B, with and without supplementary materials), and the values behind the diagnostic performance metrics (sensitivity, specificity, PPV, NPV and F1 scores with their confidence intervals) reported in the article and used to build Figure 3, Table 3 and web appendix 2. The R code used to reproduce all statistical analyses and to generate the figures is also available in the same OSF repository. This project also contains the following extended data: the PubMed search algorithms developed for each study type and validated according to the PRESS guidelines (web appendix 1), the diagnostic performance of the pipeline with and without supplementary materials (web appendix 2), the prompts used for each criterion (including abandoned variants), and the completed TRIPOD-LLM reporting checklist. Data are available under the terms of the Creative Commons Attribution 4.0 International licence (CC-BY 4.0).
