# Supplementary material for: Core open science practices in major medical journals: development of an automatized tool based on a Large Language Model
Source: Open Res Eur. 2026 Sep 7;6:326. [Version 1] doi: 10.12688/openreseurope.24615.1 (PMC13612992; doi:10.12688/openreseurope.24615.1)
Supplement: Peer review history

# Reviewer report: Reviewer response for version 1

**DOI:** 10.21956/openreseurope.26672.r80669

**Published:** 2026-09-24

**Authors:** André Luiz Appel

**Affiliations:** 1 Instituto Brasileiro de Informacao em Ciencia e Tecnologia, Brasília, Federal District, Brazil

The study is aimed at the implementation of an automated tool for the identification of open science practices across published medical articles.

 The tool was validated through the analysis of different datasets, based on 13 distinct variables or practices.

 The cited literature is limited but it reflects the originality of the proposed approach. It would be appropriate to cite literature related to the various open science practices, thereby justifying the use of a Delphi study to establish the practices being explored.

 The description of the methods employed is highly detailed and supports the study's reproducibility, including the full sharing of supporting data and the registration of the research protocol.

 The results indicate satisfactory performance for the predictive methods, demonstrating the model's viability for analyzing the implementation of open science practices in articles. They also show that variations in how articles are presented can affect the ability to extract and automatically read data; this is particularly relevant to the publishing industry, highlighting the need to adopt more widely accepted standards for metadata representation in articles.

Is the study design appropriate and does the work have academic merit?

Yes

Is the work clearly and accurately presented and does it cite the current literature?

Partly

If applicable, is the statistical analysis and its interpretation appropriate?

Yes

Are all the source data underlying the results available to ensure full reproducibility?

Yes

Are the conclusions drawn adequately supported by the results?

Yes

Are sufficient details of methods and analysis provided to allow replication by others?

Yes

Reviewer Expertise:

open science, open access journals, information science

I confirm that I have read this submission and believe that I have an appropriate level of expertise to confirm that it is of an acceptable scientific standard.

# Reviewer report: Reviewer response for version 1

**DOI:** 10.21956/openreseurope.26672.r80670

**Published:** 2026-09-22

**Authors:** Lex Bouter

**Affiliations:** 1 Vrije Universiteit, Amsterdam, The Netherlands

The manuscript reports the findings of an interesting attempt to automate the assessment of the presence of 11 open science practices with three Generative AI tools. The diagnostic accuracy of these tools is assessed in a sample of 303 articles published in one of ten major general medicine journals. It turns out that for some practices the diagnostic accuracy is acceptable to good, but for other practices it’s quite poor. The paper is well-written, and the research is performed well for the parts I’m able to judge. The technical aspects of choosing, training and applying the three GenAI models is far outside the range of my expertise, so I leave these aspects for other reviewers to comment on. A number of issues deserve attention when a revision is produced.

- I believe the authors missed an important study limitation. The articles were published in 2020-2021 and probably all written without GenAI. Currently it seems likely that in > 90% of scholarly manuscripts GenAI plays a role ranging from minor to major. That raises the question whether this would influence the diagnostic accuracy of the automated assessment of open science practices.
- To take this one step further, please also consider explicitly the risks of your admirable transparency about the GenAI models you used. That would serve unethical parties willing to produce manuscripts that pass your ‘open science exam’ better. Maybe in a follow-up study your GenAI tools could be tested for ‘game-ability’ by a ‘red team’.
- Maybe the term (open science) *indicator* would be better than the term *criteria* you currently use. For instance, mentioning *data availability on request* in the manuscript, does not at all mean that the criterion that one would actually get the data when requested will always be met. Hence my preference for the weaker term *indicator*. Please reflect on this.
- In your sampling of articles, you stratified over study designs and journals, presumably with a view to be able to detect differences in diagnostic accuracy between study designs and journals. You don’t present a power calculation supporting the idea that differences you might see would be more that random noise. Please clarify.
- You do present findings per study design, but not per journal. Please explain why.
- In the second column of table 1 you use three options to indicate inclusion in the GenAI assessments: Yes, Included and No. It’s unclear to me what the difference between the Yes and Included options are. Please explain.
- I was really surprised that the GenAI models did a poor job to identify open access publication and preprint availability. I would have expected these to be among the easiest identifiable indicators. You seem to suggest in the Discussion section that this was due to some technicality, but I found that difficult to follow. Please clarify.
- You conclude that identification of open science practices by GenAI was feasible. I would urge you to say that this is ‘feasible to some extent’. My main conclusion of the manuscript would be that automated extraction is not yet good enough to be used by journals in the manuscript assessment pipeline.
- You suggest that ‘GenAI tool plus human’ would work better than ‘Gen AI tool only’ and ‘human only’. But you did not investigate that, so I believe it would be better to rephrase the idea as a suggestion for future research.

Is the study design appropriate and does the work have academic merit?

Yes

Is the work clearly and accurately presented and does it cite the current literature?

Yes

If applicable, is the statistical analysis and its interpretation appropriate?

Yes

Are all the source data underlying the results available to ensure full reproducibility?

Yes

Are the conclusions drawn adequately supported by the results?

Partly

Are sufficient details of methods and analysis provided to allow replication by others?

Yes

Reviewer Expertise:

open science, research integrity, methodology

I confirm that I have read this submission and believe that I have an appropriate level of expertise to confirm that it is of an acceptable scientific standard, however I have significant reservations, as outlined above.

# Reviewer report: Reviewer response for version 1

**DOI:** 10.21956/openreseurope.26672.r80671

**Published:** 2026-09-11

**Authors:** Eric Jeangirard

**Affiliations:** 1 French Ministry of Higher Education and Research, Paris, France

**Summary**

 This solid study aims to test and validate the use of a local, automated pipeline to detect 13 open science practices from full-text research articles. A validation database of 303 articles was built, intended to be representative of 10 major medical journals. The local pipeline implements a RAG-type architecture, combining document parsing tools (notably GROBID 0.8.2), retrieval (BM25) and open-weights LLMs from the Llama family. The detections obtained for the 13 indicators are compared against manual extraction (with duplicate manual extraction and management of inter-annotator conflicts). The results validate the principle of the method for a subset of indicators (COI disclosure, funding statements, author contribution, data sharing, pre-registration, reporting-guideline names) with a 70-billion-parameter model (Llama 3.3-70B).

**Is the work clearly and accurately presented and does it cite the current literature?**

 Yes, with a few remarks:

 In the abstract and results, the additional computational cost of the 70B models is given as 6.6–7.5×. In the discussion, one reads "5.7 to 6.6 times higher". Is this a typo, or does it refer to something different? If the latter, it should be made explicit.

 In Results / Impact of including supplementary materials: "for either Llama 3-8B or Llama 3-70B". In Discussion / Main findings: "Including supplementary materials in the retrieval pipeline did not meaningfully change performance for any criterion when tested with Llama 3-8B and with Llama 3.3-70B." It is unclear whether the 70B model is Llama 3 or Llama 3.3.

 For COI statement detection with Llama 3-8B, the text (Tool performance) reports "F1 = 0.98 [95% CI 0.95–0.98]", whereas Figure 3 shows 0.97 (0.95–0.98).

 Regarding "the wide confidence interval around specificity and the null NPV reflect the near-total absence of true negatives for this criterion (COI disclosure was positive in ~100% of articles) rather than a genuinely unstable estimate": would it not be preferable to report these values as "not estimable"?

 The RRID links (http://scicrunch.org/resources/Any/search?q=SCR_001905 and SCR_019186) return an error. Moreover, there is no consensus on using RRIDs to identify software. Why not Wikidata, or even a SWHID (https://www.swhid.org/)?

**Is the study design appropriate and does the work have academic merit?**

 Yes

 The study design is very clear and well suited to the question, with duplicate manual extraction and reconciliation, and the comparison of different sizes of local models.

 However, for some of the 13 open science practices, it is not entirely clear what is expected from the pipeline (nor, in fact, from manual extraction), when the practice is strongly tied to a use "external" to the publication itself, notably open access status, ORCID, and publication of results within one year. For the latter, estimating the publication delay requires dates (of publication, of sharing) that belong to external metadata (registries). The same applies to the presence of ORCID identifiers (publication metadata).

 Regarding the decision to exclude the criterion "Reporting the use of persistent identifiers when sharing data/code/materials": one could nevertheless expect such a PID to be mentioned whenever sharing occurs, since it greatly facilitates retrieval, much like a DOI for publications.

**Are sufficient details of methods and analysis provided to allow replication by others?**

 Yes

 It would have been useful to report inter-annotator agreement before reconciliation. The only related mention is: "the adjudication process itself revealed that a sizable fraction of initial annotations for some criteria (notably data sharing, where roughly a third of audited 'data sharing' labels were corrected) reflected disagreement about definitions rather than oversight."

**If applicable, is the statistical analysis and its interpretation appropriate?**

 Do the authors have hypotheses explaining why the 8B model performs better than the 70B models on COI and pre-registration? This is surprising. A chain-of-thought approach might help understand why the 70B model can be mistaken when the 8B is right.

**Are all the source data underlying the results available to ensure full reproducibility?**

 Yes

**Are the conclusions drawn adequately supported by the results?**

 Regarding the final "recommendation" of Llama 3.3-70B, more nuance would be prudent. Compared with the 8B model, the confidence intervals overlap substantially, for a high computational cost. It would be possible to weigh raw "performance" (to be put into perspective, since some criteria are poorly suited to the method and the gold standard remains uncertain) against computational cost/time, depending on the goals of those who would deploy such a pipeline.

 The generalisability of the results could be discussed in more depth, even if this goes beyond the initial research question:

 - languages other than English (Latin alphabet or not, etc.);

 - disciplines other than medicine;

 - criteria other than the 13 selected ; on this point, the RAG architecture is particularly flexible and could be adapted to other criteria; this would be worth highlighting.

 - ability of the pipeline to detect more than a yes/no indicator, but to identify some research outputs (shared data for example)

Is the study design appropriate and does the work have academic merit?

Yes

Is the work clearly and accurately presented and does it cite the current literature?

Yes

If applicable, is the statistical analysis and its interpretation appropriate?

Yes

Are all the source data underlying the results available to ensure full reproducibility?

Yes

Are the conclusions drawn adequately supported by the results?

Yes

Are sufficient details of methods and analysis provided to allow replication by others?

Yes

Reviewer Expertise:

scholarly communication, open science monitoring

I confirm that I have read this submission and believe that I have an appropriate level of expertise to confirm that it is of an acceptable scientific standard.
